# Medication-overuse headache: an update

**DOI:** 10.1186/1129-2377-16-S1-A46

**Published:** 2015-09-28

**Authors:** Paola Sarchielli

**Affiliations:** Clinica Neurologica, Azienda Ospedaliero - Universitaria di Perugia, Perugia, Italy

Medication-overuse headache (MOH) is a chronic daily headache evolving from an episodic primary headache (mainly migraine) due to overuse of one or more classes of migraine abortive medication.

The development of MOH is associated with both overuse of one or more medication and behavioral predispositions.

MOH causes a significant decline in the quality of life and reduces functioning in patients affected. In the same MOH patients, the presence of psychopathological disturbances may be a predictor of relapse and poor response to treatment.

In a recent study of our group, we found that MOH patients had a more complex profile of psychiatric comorbidity compared to episodic migraine (EM) patients. Furthermore, clinically relevant obsessive-compulsive disturbances for abused drugs assessed by Yale-Brown Obsessive Compulsive Scale Y-BOCS, appeared to be more represented in the MOH group, while the prevalence of this trait in the EM group was comparable to that of healthy controls (unpublished results).

Management of MOH represents a difficult challenge for clinicians and headache experts, particularly because of the high percentage of relapse after a successful withdrawal treatment.

This can be addressed if the patient is followed over a prolonged period of time with a combination of prophylactic pharmacotherapy and use of abortive medication with minimal risk of MOH, avoiding previously overused medications.

With the aim to verify the efficacy and safety of sodium valproate in the short-term treatment of MOH, we recently carried out a multicentre study (SAMOHA study) which demonstrated a superiority of the drug compared to placebo after detoxification[1].

In an ancillary study of SAMOHA, analysing responders and non responders to detoxification and advice to withdraw a benefit was excluded in patients with a long history of MOH[2].

In a further ancillary research we found a significant correlation between MOH relapse and the total MSQ score, the Role Preventive and the Emotional Function sub-scales, suggesting a poorer quality of life in non responders[3].

Recent evidence suggests an involvement of genetic factors in predisposition to medication overuse. In a recent study of our group involving a subsample of MOH patients enrolled in the SAMOHA study, we sequenced all exons, intron/exon junctions and 3'-5'UTR regions of HDAC3 gene which had been implicated in excessive medication consumption in MOH patients. Univariate analysis showed that the G allele of the exonic SNP rs2530223 was significantly associated with the number of acute medications/month used and with the number of days/month in which acute medication were used (unpublished results).
